# Neurocognitive and emotional benefits of choir singing and their mediating factors across adulthood

**DOI:** 10.1007/s40520-025-03187-1

**Published:** 2025-09-04

**Authors:** Nella Moisseinen, Alli Haavisto, Silja Heimala, Noelia Martínez-Molina, Boris Kleber, Aleksi J. Sihvonen, Teppo Särkämö

**Affiliations:** 1 Cognitive Brain Research Unit, Centre of Excellence in Music, Mind, Body and the Brain, Department of Psychology, Faculty of Medicine, University of Helsinki, Helsinki, Finland; 2 Centre for Brain and Cognition, Department of Information and Communication Technologies, University Pompeu Fabra, Barcelona, Spain; 3 Center for Music in the Brain, Department of Clinical Medicine, Aarhus University & The Royal Academy of Music Aarhus/Aalborg, Aarhus, Denmark; 4 Centre for Clinical Research, School of Health and Rehabilitation Sciences, University of Queensland, Brisbane, Australia; 5 Department of Neurology, Helsinki University Hospital, Helsinki, Finland

**Keywords:** Ageing, Music, Episodic memory, Verbal fluency, Mood, Quality of life (QOL)

## Abstract

**Background:**

Our understanding on how cognitive and socioemotional well-being factors interact throughout adulthood has increased remarkably over the past decades, encouraging the use of cognitively engaging leisure activities, such as music, to promote healthy ageing. Choir singing has attracted particular interest in this regard with its established benefits on socioemotional well-being. Outside the clinical context, however, the cognitive and well-being effects induced by musical activities are often studied separately, leaving it unclear to what extent they interact in contributing to healthy ageing.

**Aims & methods:**

Using a balanced sample (*N* = 95) of healthy adults (aged 21–88 years) with neuropsychological test data (verbal fluency, processing speed, executive function, working memory) and questionnaire data (depression and quality of life, QOL), the present study mapped the benefits of choir singing (duration and frequency) on cognitive functions and well-being and their mediating mechanisms across adulthood.

**Results:**

Choir singing frequency was associated with reduced depressive symptoms, which also mediated effects with QOL. Lifetime duration of choir singing was associated with enhanced episodic memory and verbal fluency, with a mediating effect of semantic verbal fluency on the relationship between choir singing and episodic memory.

**Discussion & conclusions:**

These findings convey the co-occurrence of singing-associated benefits and characterise shared mechanisms by which these effects interact in promoting healthy ageing.

## Introduction

The brain’s unique ageing trajectory unfolds throughout adulthood, following the net influence of accelerating and mediating factors, such as ageing-related sensory deficits [1, 2], genetic determinants, and lifestyle [3]. The global demographic shift towards an older population [4] has increased interest towards cognitively engaging and ecologically valid leisure activities, including arts, to promote healthy ageing across nations. Increasing evidence supporting the efficacy of music, in particular, is now advocated in this context by healthcare organizations including WHO [5] and Global Council on Brain Health [6].

Crucially, music can support verbal fluency and auditory-linguistic skills [7–9], episodic memory and learning [7, 10, 11], working memory [7, 10], processing speed, and executive function [10] at older age. The mechanisms underlying these benefits remain less clear, however, especially with respect to the continuum of cognitive decline across these domains as well as their mutual interactions in everyday cognition.

One well-known example in ageing literature is episodic memory, where performance can be predicted with performance at earlier time points [12, 13] but also with performance in (semantic) verbal fluency. In the clinical setting, verbal fluency tasks can be used to predict progression from normal ageing to mild cognitive impairment (MCI) and Alzheimer’s type dementia [14, 15]. Considering that music activities have been associated with enhancements in multiple functions, episodic memory and verbal fluency included, understanding potential mediating effects between them would provide valuable information on how music can be used to support healthy ageing.

A promising candidate would be singing, which naturally involves lexical-semantic mapping while supporting episodic memory [16] and acting as an effective mnemonic cue [17, 18]. Although singing, especially choir singing, has established well-being effects based on qualitative evidence [19–23], it has remained underrepresented in neurocognitive research literature, especially with respect to dose effects of singing at amateur level [7, 24, 25], and the decades-long continuum of ageing in adulthood. To fill these gaps in current literature, we systematically mapped the associations of choir singing experience and cognitive performance and well-being measures, as well as their mediating effects, across the adult lifespan using a balanced sample of cognitively intact, healthy adults (*N* = 95; aged 21–88 years). Further, to learn about the influence of training factors (dose effects) and their potential overlap across the full age range, outcome measures were studied with respect to lifetime duration as well as lifetime maximal and present frequency of singing using stepwise linear regression and mediation models.

## Methods

### Participants

One hundred volunteers (55 female), aged 21–88 years (mean 49.2, SD 17.5), were recruited to participate in a research project studying the associations of choir singing experience with well-being and cognitive function as well as brain structure [26]. To maintain uniformity across methods, the present study used the same sample as a neuroimaging study belonging to the same project [26], leading to a final sample size of *N* = 95. All participants were right-handed native Finnish-speakers (4 bilingual) with no diagnosis of a hearing impairment, language or neurological disorder, cognitive decline, or dementia. The recruitment process controlled for the main demographic characteristics of the sample to ensure a balanced representation of different ages across the desired age range (20–90 years) as well as gender and choir singing experience both within the full sample and within the three age groups (young, aged 20–39 years; middle-aged, 40–59 years; older, 60 years and above).

As the present study focused on amateur-level musical activity, no professional background in music was allowed. Choir singing was required to be the primary active musical hobby for participants who reported having choir singing experience in adulthood, with a minimum frequency of 1 weekly hour of choir singing for the past year or more. Persons with no choir singing experience or those with some experience in childhood (e.g., elementary school choir) but none in adulthood were allowed in the non-singer group.

All participants provided a written informed consent to participate and to the use of their data for the scientific purposes of this study. The study was conducted in accordance with the Helsinki Declaration and approved by the European Research Council Executive Agency (ERCEA) and the University of Helsinki Ethical Review Board in the Humanities and Social and Behavioural Sciences.

### Measures and procedure

Participants filled out questionnaires assessing recent depressive symptoms (Center for Epidemiological Studies: Depression, CES-D [27]) and experienced quality of life (QOL), comprising designated subscales for Overall, Physical, and Psychological QOL, Social relationships, and Environment (WHOQOL-BREF [28], which were used to assess subjective well-being.

In addition, participants reported the durations (years) and frequencies (weekly hours) of present and past musical hobbies. As the theoretical maximum of lifetime duration is directly limited by age, the duration scores do not scale between the lower and upper ends of the age range of the present sample (21–88 years). Specifically, this leads to age-dependent ranges for the same regressor, meaning that the results from the correlational models represent a mixture of scales across the full age range. To improve mutual scalability of results across the age range and between the age-specific subgroups, age-associated differentiation of the raw duration score was corrected by transforming the years of active participation into age-adjusted scores, specifically, % of age. The frequency of choir singing as well as other musical hobbies were assessed by (i) the most active period (average hours/week), i.e., lifetime maximum, and (ii) the present level of activity (average hours/week).

Neuropsychological assessments (duration approximately 1.5 h) were conducted in a quiet room at the laboratory of Cognitive Brain Research Unit (CBRU), University of Helsinki, by a graduate student trained by a licensed psychologist; the psychologist was also consulted upon any uncertainties in scoring. The neuropsychological test battery assessed verbal fluency (phonemic: letter S, semantic: animals), processing speed (Wechsler Adult Intelligence Scale, 4th edition [WAIS-IV], processing speed subtests: Symbol search, Digit symbol coding; [29]), working memory (WAIS-IV, working memory subtests: Digit span, Arithmetic), immediate and delayed episodic memory (Wechsler Memory Scale, 3rd edition [WMS-III], auditory immediate and delayed recall subtests: Logical memory, Word lists; [30]), and executive function (Trail making tests [TMT] A and B from the flexible attention test (FAT) developed at the Finnish Institute of Occupational Health [31]; Simon task [32, 33]). Tests were carried out in the traditional pencil-and-paper format apart from tests of executive function, in which a touch screen laptop computer was used for higher accuracy in time observations. Here, response time difference scores were calculated as TMT B minus A and Simon task incongruent minus congruent to obtain indices for shifting and inhibition, respectively. Given the large age range of the sample and the specific aim to study ageing effects, raw instead of standardised scores were used in analysis, and summed into thematic composite scores for each domain.

### Analysis

#### Linear regression models

To learn about the dose effects of training factors, the associations of choir singing with well-being (CES-D, WHOQOL-BREF) and cognitive performance (verbal fluency, processing speed, working memory, episodic memory [immediate and delayed recall], and executive function) scores were tested with linear stepwise regression models in SPSS (IBM SPSS Statistics, version 29). As correlational music studies are subject to population bias through natural disposition for music [34] (pp. 367–377), the regression models were carried out for (i) a subgroup of non-professional choir singers with various degrees of singing experience (*N* = 53), to learn about potential dose effects even in the presence of such bias, and for (ii) the full sample (*N* = 95) including also individuals with no choir singing experience, to see whether effects within choir singers would generalise to the full sample or be lost in noise, in which case the effect would be non-unique to choir singing (or singers). To address any relevant age-specificity within effects in choir singers, the primary sources of each surviving result were also determined by assessing the effects among (iii–v) three age-specific subgroups (young: 20–39 years, middle-aged: 40–59 years, older: 60 and above).

Each stepwise model involved all training factors (choir singing duration [% of age], lifetime maximal and present frequency) to learn whether they may contribute in parallel, including also age, education (years), and other musical hobbies (duration, maximal and present frequency of solo singing and instrument playing) as covariates. The results were inspected for violations in normality, independence of errors, and homoscedasticity. Outliers were identified with Mahalanobis distance, Cook’s distance, and centred leverage, removing any case falling outside at least two out of the three distances. Finally, the alpha level was adjusted to 0.01 to account for multiple comparisons across the five models per dependent factor (Bonferroni).

#### Mediation models

To avoid testing for random or ‘nearby’ mediation effects [35, 36], simple mediation analyses were limited to theory-informed models to test for basic multivariable effects among and between well-being and cognitive performance.

Mediation analyses were conducted using the PROCESS macro [37], version 4.2, running under SPSS (IBM SPSS Statistics, version 29), with 10 000 bootstrapped samples. Here, a mediation is proposed to exist when zero falls outside the lower and upper levels of the mediator-specific 95% bootstrap confidence interval (CI for a detailed description on this approach, see [37], pp. 99–107). Each mediation model controlled for the same factors which showed a significant contribution to the initial regression effect serving as the motivation for testing mediation. Since mediation was tested selectively, any initial regression effect falling below uncorrected *α* = 0.05 was considered for mediation models.

## Results

### Sample

The final sample (*N* = 95) was matched with a neuroimaging study belonging to the same research project [26]. In the neuropsychological assessments, a technical failure in the Trail Making Test and Simon task further reduced the sample size by two participants, leading to a final sample size of *N* = 93 for analyses on executive function. No data outliers were found.

The characteristics of the sample are summarised in Table 1. On average, choir singers (*N* = 53) reported having been engaged in amateur-level choir singing for approximately 35% of their lives, involving 2–10 weekly hours at maximum and 1–8 weekly hours at present. From non-singers (*N* = 42), none had sung in a choir in adulthood, while 10 reported some choir singing experience in childhood (e.g., elementary school choir) with a lifetime duration of 3–16% involving 1–4 weekly hours.

**Table 1:** Characteristics of the sample including demographic information and musical experience, well-being and cognitive performance. Associations between choir singing and well-being as well as cognitive performance scores were tested for the full sample and separately for participants reporting at least some choir singing experience in adulthood. Information about non-singers (persons with no choir singing experience in adulthood) provided for comparison. All values reported as mean (SD), min–max, unless otherwise specified

	All (N = 95)	Choir singers (N = 53)	Non-singers (N = 42)
**Demographic**			
Age	49.2 (17.6), 21–88	51.4 (17.8), 22–88	46.4 (17.1), 21–85
Gender (female/male/other)	50/45/0	27/26/0	23/19/0
Education years	16.6 (4.3), 2–34	16.6 (4.2), 2–22	16.6 (4.4), 7–34
**Musical background (amateur level)**			
***Choir singing***			
% of life active	20.5 (24.1), 0–79	35.4 (23.0), 3–79	1.8 (3.7), 0–16
Maximal frequency (hours/week)	2.7 (2.7), 0–10	4.6 (2.1), 2–10	0.4 (0.9), 0–4
Present frequency (hours/week)	1.8 (2.0), 0–8	3.1 (1.7), 1–8	0
***Solo singing***			
% of life active	9.4 (19.7), 0–94	11.8 (19.2), 0–87	6.4 (20.1), 0–94
Maximal frequency (hours/week)	1.7 (3.2), 0–15	2.1 (3.3), 0–15	1.1 (3.1), 0–15
Present frequency (hours/week)	0.5 (1.2), 0–8	0.6 (1.1), 0–5	0.4 (1.5), 0–8
***Playing a musical instrument***			
% of life active	22.8 (27.3), 0–87	32.2 (29.3), 0–87	10.9 (19.1), 0–81
Maximal frequency (hours/week)	2.8 (4.0), 0–20	3.7 (4.5), 0–20	1.7 (3.0), 0–15
Present frequency (hours/week)	0.6 (1.4), 0–10	0.7 (1.5), 0–10	0.5 (1.3), 0–7
**Depressive symptoms**^1^			
Total	10.0 (6.4), 0–30	9.7 (6.2), 0–29	10.4 (6.7), 0–30
**QOL**^2^			
Overall	16.9 (2.2), 10–20	17.2 (1.9), 12–20	16.5 (2.5), 10–20
Physical	16.9 (2.1), 9–20	17.0 (2.0), 11–20	16.9 (2.2), 9–20
Psychological	16.2 (2.0), 10–20	16.3 (2.0), 10–20	16.0 (2.1), 11–20
Social relationships	16.0 (2.8), 8–20	15.9 (3.0), 8–20	16.2 (2.5), 11–20
Environment	17.7 (1.7), 11–20	18.0 (1.7), 11–20	17.3 (1.8), 12–20
**Cognitive performance**			
Verbal fluency^3^	45.8 (11.0), 22–78	46.0 (11.3), 26–78	45.6 (10.7), 22–72
Processing speed^4^	97.5 (24.6), 39–143	98.0 (24.9), 52–142	96.8 (24.4), 39–143
Working memory^5^	43.9 (7.1), 27–60	44.8 (6.6), 27–60	42.7 (7.5), 28–59
Episodic memory, immediate^6^	75.2 (13.7), 42–112	75.6 (14.2), 42–112	74.6 (13.3), 53–100
Episodic memory, delayed^7^	34.3 (9.4), 9–56	35.0 (9.5), 9–56	33.5 (9.2), 16–52
Executive function^8,9^	26.8 (22.1), −5.8–10.7	24.0 (21.0), −3.4–10.7	30.4 (23.2), −5.8–9.6

^1^Center for Epidemiological Studies: Depression (CES-D). ^2^WHOQOL-BREF. ^3^Phonemic (letter S) + semantic (animals); unique items produced in 60 s in each. ^4^WAIS Symbol search + WAIS Digit symbol coding**.** ^5^WAIS Digit span + WAIS Arithmetic. ^6^WMS Logical memory story A + B (1.) + B (2.) immediate + WMS Word lists immediate. ^7^WMS Logical memory story A + B delayed + WMS Word lists delayed. ^8^TMT (B-A) + Simon task (incongruent minus congruent); sum of response time differences in seconds. ^9^NB. Sample size reduced by two participants due to a technical failure during acquisition, leading to a full sample size of *N* = 93 participants with *N* = 53 singers and* N* = 40 non-singers

As intended by a priori control during recruitment, age and gender were not associated within the full sample (*N* = 95) nor within choir singers (or non-singers). Further, choir singers (included in subgroup and full-sample correlational models) and non-singers (included in full-sample correlational models) did not differ across demographic background factors (age, gender, education). Consistent with the demographic characteristic of the Finnish population [38, 39], higher age was negatively associated with education years [*r*_*s*_(93) =  − 0.272, *p* = 0.008], with middle-aged participants reporting more years on average. From choir singing measures, duration (% of age) and maximal or present frequency (hours / week) were not associated with age, gender, or education (years). By contrast, duration and present frequency of solo singing [*r*_*s*_(93) =  − 0.251, *p* = 0.014; *r*_*s*_(93) = -0.362, *p* < 0.001, respectively] and instrument playing [*r*_*s*_(93) =  − 0.262, *p* = 0.010; *r*_*s*_(93) = -0.205, *p* = 0.046, respectively] were all negatively associated with age, suggesting that younger participants were more active in other musical activities.

### Choir singing and well-being

Sample characteristics and details on regression results for well-being measures are provided in Table 1 and Table 2, respectively. Among choir singers, the present frequency of choir singing (hours/week) was negatively associated with depressive symptoms with no significant contribution from other factors in the stepwise model. A similar trend was found across the full sample (*p* = 0.025), however, a violation of the independence of errors was observed, and the model did not survive correction for multiple comparisons at *α* = 0.01 (Bonferroni). No other effects for depression were found.

**Table 2:** Results from stepwise multiple regression for depressive symptoms, QOL, verbal fluency (semantic and phonological) as well as episodic memory (immediate and delayed recall) in the full sample and choir singers. Results from secondary analyses within age-specific subgroups of choir singers reported when applicable. Alpha levels corrected for multiple comparisons at α = .010 (Bonferroni) for each dependent factor. Stepwise models found no significant associations for choir singing and other domains of QOL or other neuropsychological test scores (processing speed, working memory, and executive function; not reported)

	Model	Coefficient(s)					
	*df*	*Adj. R*^*2*^	*F*	*p*	*Std. β*	*t*	*p*
**Depressive symptoms**^1^							
***Full sample***							
Present freq. of choir singing	1, 93	.053	5.192	.025	−.230	−2.278	.025
**Choir singers**							
Present freq. of choir singing	1, 51	.161	10.955	.002	−.421	−3.310	.002
**QOL (WHOQOL** **overall)**^2^							
***Full sample***							
Present freq. of choir singing	1, 93	.059	5.824	.018	.243	2.413	.018
***Choir singers***							
Present freq. of choir singing	1, 51	.070	4.894	.031	.296	2.212	.031
**QOL (WHOQOL psychological)**^2^							
***Choir singers***							
Present freq. of choir singing	1, 51	.086	5.881	.019	.322	2.425	.019
**Verbal fluency (animals + letter S)**^3^							
***Full sample***							
Choir singing duration (% of age)	2, 92	.174	10.900	< .001	.198	2.112	.037
Age					−.403	−4.291	< .001
***Choir singers***							
Choir singing duration (% of age)	2, 50	.335	14.102	< .001	.260	2.298	.026
Age					−.533	−4.715	< .001
**Episodic memory (immediate)**^4^							
***Full sample***							
Choir singing duration (% of age)	2, 92	.371	28.717	< .001	.222	2.713	.008
Age					−.593	−7.235	< .001
***Choir singers***							
Choir singing duration (% of age)	2, 50	.360	15.598	< .001	.248	2.230	.030
Age					−.561	−5.049	< .001
**Episodic memory (delayed)**^5^							
***Full sample***							
Choir singing duration (% of age)	3, 91	.403	22.150	< .001	.257	3.222	.002
Age					−.460	−5.325	< .001
Education (years)					.265	3.076	.003
***Choir singers***							
Choir singing duration (% of age)	3, 49	.389	12.023	< .001	.282	2.591	.013
Age					−.398	−3.252	.002
Education (years)					.288	2.350	.023

^1^Center for Epidemiological Studies: Depression (CES-D). ^2^WHOQOL-BREF. ^3^Phonemic (letter S) + semantic (animals); unique items produced in 60 s in each. ^4^WMS Logical memory story A + B (1.) + B (2.) immediate + WMS Word lists immediate. ^5^WMS Logical memory story A + B delayed + WMS Word lists delayed

Similarly, among choir singers, present frequency of choir singing predicted psychological and overall QOL at trend level (*p* = 0.019; *p* = 0.031, respectively). For overall QOL, a trend was also observed for the full sample (*p* = 0.018) which, however, showed a violation of the normality assumption and should thus be interpreted with caution. The association between the present frequency of choir singing and psychological QOL was not significant in the full sample (*p* = 0.055). Crucially, none of the QOL models survived correction for multiple comparisons. No other effects for QOL were found.

**Fig. 1:**
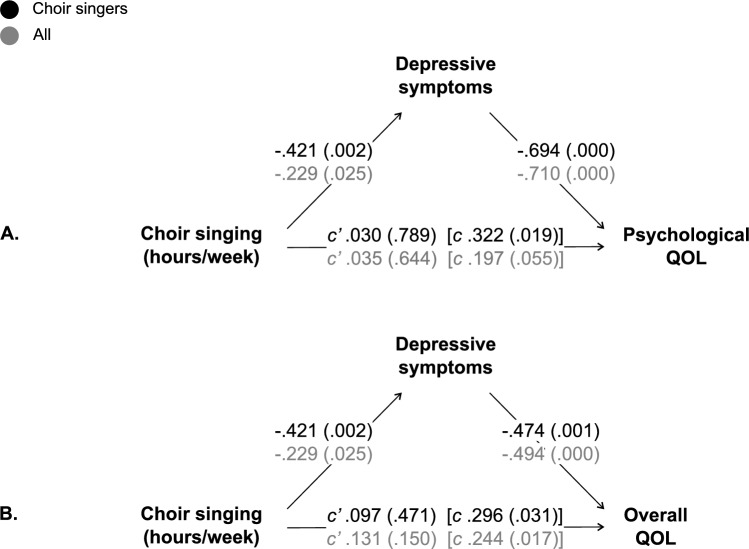
Mediation results for the present frequency of choir singing (hours/week) and QOL (**A** psychological QOL; **B** overall QOL) via self-reported depressive symptoms, with the direct effects of choir singing (c’), using 10 000 bootstrapped samples**.** Total effects of choir singing (c) provided for comparison. Results for choir singers and the full sample displayed in black and grey, respectively. Each result reported as the standardised coefficient (p value)

As previous evidence suggests that depressive symptoms predict QOL [40], QOL was selected as the outcome measure for mediation. Details on the mediation results are provided in Table 3 and Fig. 1. Among choir singers, the positive associations between present choir singing frequency and psychological QOL (Fig. 1A) as well as overall QOL (Fig. 1B) were mediated by the reduced depressive symptoms. Supplementary analyses for the full sample suggested weaker mediation effects for both psychological and overall QOL. However, the full-sample results should be interpreted with caution as per violations described above.

**Table 3:** Mediation results for choir singing (X; present frequency as hours/week, lifetime duration as % of age) and well-being as well as cognitive factors (Y) via respective mediators (M), for the full sample and choir singers using 10 000 bootstrapped samples. Results where 0 does not fall within the CI (95%) are considered statistically significant.

Y, X	M	Indirect	SE	CI (95%)
**QOL** **(WHOQOL overall)**^1^	**Depressive symptoms**^2^			
***Full sample***				
Present freq. of choir singing	CES-D (total)	.128	.058	.013 – .241
***Choir singers***				
Present freq. of choir singing	CES-D (total)	.230	.089	.084 – .437
**QOL** **(WHOQOL psychological)**^1^				
***Full sample***				
Present freq. of choir singing	CES-D (total)	.166	.073	.018 – .310
***Choir singers***				
Present freq. of choir singing	CES-D (total)	.354	.119	.153 – .630
**Episodic memory (immediate)**^3^	**Verbal fluency**^4^			
***Full sample***				
Choir singing duration (% of age)	Composite	.024	.018	−.001 – .069
Choir singing duration (% of age)	Phonemic (letter S)	.005	.001	−.001 – .029
Choir singing duration (% of age)	Semantic (animals)	.027	.018	.003 – .071
***Choir singers***				
Choir singing duration (% of age)	Composite	.044	.034	−.001 – .127
Choir singing duration (% of age)	Phonemic (letter S)	.005	.015	−.018 – .043
Choir singing duration (% of age)	Semantic (animals)	.060	.041	.002 – .159
**Episodic memory (delayed)**^5^				
***Full sample***				
Choir singing duration (% of age)	Composite	.013	.011	−.001 – .039
Choir singing duration (% of age)	Phonemic (letter S)	.001	.001	−.001 – .012
Choir singing duration (% of age)	Semantic (animals)	.016	.012	.000^6^ – .044
***Choir singers***				
Choir singing duration (% of age)	Composite	.029	.023	−.001 – .087
Choir singing duration (% of age)	Phonemic (letter S)	.003	.001	−.013 – .027
Choir singing duration (% of age)	Semantic (animals)	.042	.026	.004 – .103

^1^WHOQOL-BREF. ^2^Center for Epidemiological Studies: Depression (CES-D). ^3^WMS Logical memory story A + B (1.) + B (2.) immediate + WMS Word lists immediate. ^4^Composite score represents phonemic (letter S) + semantic (animals); unique items produced in 60 s in each task. ^5^WMS Logical memory story A + B delayed + WMS Word lists delayed. ^6^Exact value: .0001

### Choir singing and cognitive function

Sample characteristics and regression results for cognitive performance measures are provided in Table 1 and Table 2, respectively. Duration of choir singing experience (% of age) predicted immediate episodic memory performance together with age within choir singers as well as in the full sample. Duration of choir singing experience also predicted delayed episodic memory performance within choir singers, again with a similar effect in the full sample. Delayed episodic memory performance was also partially explained by age and education years. Post hoc analyses in the age-specific subgroups did not show significant results with either memory score. Finally, the duration of choir singing experience predicted verbal fluency together with age within choir singers as well as in the full sample. Verbal fluency was also partially explained by age, but the result did not survive in any of the age-specific subgroups.

**Fig. 2:**
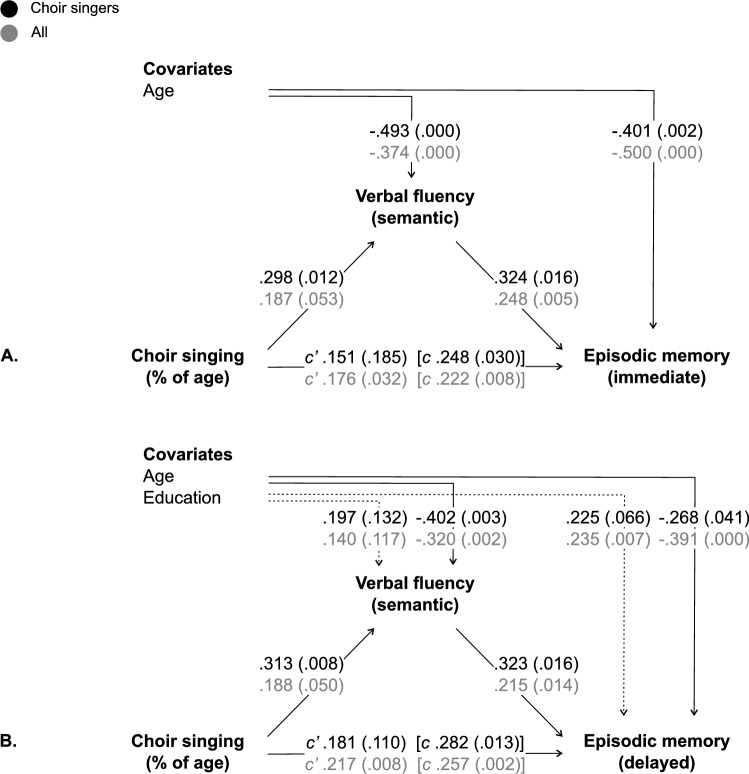
Mediation results for the lifetime duration of choir singing (% of age) and episodic memory (**A** immediate recall; **B** delayed recall) through semantic verbal fluency (animals), with the direct effects of choir singing (c’), using 10 000 bootstrapped samples. Total effects of choir singing (c) provided for comparison. Results for choir singers and the full sample displayed in black and grey, respectively. Each result reported as the standardised coefficient (p value)

Based on previous evidence, the significance of mediation was tested for episodic memory via verbal fluency. Since previous evidence suggests differential associations of phonemic versus semantic fluency with episodic memory in ageing [14, 15], mediation analyses via verbal fluency were carried out for the composite score (phonemic + semantic) as well as separately for each of its subcomponents. Details on the mediation results are provided in Table 3 and Fig. 2. Mediation models on the composite score of verbal fluency fell outside significance; this trend was repeated in phonemic fluency. By contrast, mediation through semantic fluency onto episodic memory was significant for both immediate (Fig. 2A) and delayed (Fig. 2B) episodic memory scores in both choir singers and, with smaller indirect effects, in the full sample.

## Discussion

The present study mapped the associations of choir singing experience (duration [% of age], lifetime maximal frequency and present frequency [hours/week]), with self-reported well-being measures in depressive symptoms and QOL as well as neuropsychological test scores in verbal fluency, processing speed, working memory, episodic memory, and executive function, while controlling for the effects of age, education, and other musical hobbies. Results from linear stepwise regression analyses suggested, first, that the present frequency of choir singing is associated with reduced depressive symptoms, which mediated effects in psychological and overall QOL. Second, the duration of choir singing experience was associated with enhanced immediate and delayed episodic memory, together with age and education years, and with enhanced verbal fluency together with age. Enhancements in semantic verbal fluency mediated the effects in both episodic memory scores.

### Psychological well-being associated with choir singing

Qualitative evidence suggests that choir singing promotes psychological well-being by enhanced emotional expression through singing, reduced negative and increased positive affect, as well as experiences of ease and uplift [19, 21] (for systematic reviews involving clinical populations, see also [41, 42]). Results from randomised controlled trials (RCTs) suggest a direct [20, 43] or indirect [22] causal relationship, where an interplay of affect and other components of psychological well-being produces the overall effect (see also [44, 45]; for contrasting results, see [46, 47]). Previous reports also note that psychological well-being may begin to decline shortly after singing is discontinued, indicating the importance of active engagement [20]. Following the end of a group-singing-based RCT, the interviewees of Skingley and colleagues [43] also expressed regret regarding the end of the singing program along with concerns about succeeding changes in mood; resuming singing activities, on the other hand, was expected to restore positive mood.

Apart from reduced depressive symptoms within choir singers, the present well-being effects remained at trend level. Given that the present effects align well with previous findings, the failure of the present results to survive correction for multiple comparisons may partially owe to the limited sample size and naturalistic bias from a cross-sectional design. As previous work is largely based on data from ageing populations, however, observing trends towards similar effects across a notably larger age range (from early 20s up) suggests that these benefits may extend throughout adult life. In favour of this idea, age did not appear a significant predictor in the models, nor did the effects reach significance for the age-specific subgroups. While the latter could be due to significantly smaller sizes of the age-specific subsamples as compared to previous works (e.g., *N* = 258 in [20]), it should be noted that the overall predictive outcomes for choir singing, especially with respect to QOL measures, were rather modest. Given that the associations with well-being measures were not strengthened but further reduced through all full-sample models, the present pattern of effects seems to suggest that choir singing is a source of psychological well-being, yet that its effects are not highly unique but comparable to other sources of well-being in the general population. This corroborates findings from comparative studies, which tentatively suggest that choir singing is associated with benefits on psychological well-being comparable to other leisure activities, such as team sports [48, 49].

Finally, the present results suggested a positive mediation effect from ongoing choir singing frequency onto psychological and overall QOL via reduced depressive symptoms. Music is considered to improve treatment outcomes for depression [50] while providing a tool for mood regulation [51] and evoking rich emotional responses through, for instance, the dopaminergic reward system, stress hormone cortisol, and social hormone oxytocin [52–54]. Although increasing evidence suggests that choir singing is associated with better psychological well-being [43], also when compared to solo singing [23], this is not systematically reflected in biomarkers such as cortisol [55, 56]. It is recommended that future studies should address the mechanisms by which choir singing (or other forms of music) could contribute to psychological well-being, keeping in mind the variability in immediate versus long-term effects, as well as considering interactions between biomarkers [53] and the various sources of well-being accompanying choir singing in daily life.

### Music and linguistic-semantic processing

Music carries meaning through the complex interactions between harmony, rhythm, intensity, and other features [57], where sung lyrics can also convey the semantic contents of natural language. The present results suggested a positive association of choir singing duration with verbal fluency. Consistent with the linguistic-semantic demands of these tasks [58], our earlier neuroimaging study using the same sample [26] found duration-associated enhancements of the microstructure (as shown by quantitative anisotropy, QA) of the arcuate fasciculus and inferior fronto-occipital fasciculus (IFOF), which are key pathways of the dorsal and ventral processing streams of the widespread language network [59]. Together, this pattern of results corroborates and extends previous findings showing transfer effects from singing to verbal fluency in healthy ageing [8, 46] and Alzheimer’s disease [60], suggesting that singing-induced enhancements on fluency tasks may be associated with enhanced structural connectivity of the language network. While the complex demands [58] of verbal fluency tasks suggests that singing-associated enhancements can stem from more than one origin, the lexical-semantic mapping along the ventral stream, including the far-branching IFOF (cf. [61, 62]), especially, offers a promising target for future works aiming to establish direct associations between behavioural and neural outcomes.

Despite established transfer effects from music to language, especially with respect to auditory-acoustic processing [63, 64], lexical-semantic mapping can only occur in the presence of words. While the present analyses with verbal fluency used a composite score (semantic + phonemic), comparisons between its subscores falling outside the scope of this behavioural mapping study, we note that the supplementary mediation analyses with episodic memory and verbal fluency showed positive associations between choir singing duration and semantic verbal fluency within choir singers (Fig. 2; see also [8, 60]). By contrast, instrumental activities have been repeatedly associated with phonemic but not semantic fluency [7, 65]. This asymmetry motivates direct comparisons between singing and instrumental activities for future works. Specifically, it would be highly interesting to learn how potential differences might be associated with music-induced benefits on memory and executive processing, given their associations with age-sensitive verbal fluency tasks [58].

### Singing, ageing and episodic memory

Consistent with present findings, previous evidence suggests a strong association between music and episodic memory. Music can act as a mnemonic cue [17, 18], reduce the risk of dementia [11, 66], and improve episodic memory performance in healthy ageing [10, 16, 24] as well as in early stages of dementia [60, 67]. Further, music-evoked episodic memories are often long preserved in progressive memory diseases such as Alzheimer’s disease (for suggested neural mechanisms, see [68]).

Episodic memory performance typically echoes earlier performance in the long continuum of ageing [12]. In the clinical context, semantic verbal fluency is also identified as an independent predictor of episodic memory performance and of progression from normal ageing to mild cognitive impairment [14, 15] and to Alzheimer’s type dementia [14, 69]. Consistent with this, the present results suggested that enhanced episodic memory performance was positively mediated by semantic verbal fluency. While the nature of the association between episodic memory and semantic verbal fluency remains less clear, it is too early to speculate whether the present results indeed stem from the same origin; testing for these effects in a longitudinal design would provide a better basis for estimating mediation as well as its origin. It is noteworthy, however, that the present mediation effect was significant across adulthood, suggesting that this mechanism could explain the benefits of singing on episodic memory throughout the long continuum of ageing. Indeed, parallel enhancements from singing on episodic memory and semantic fluency have even been reported in mild Alzheimer’s disease [60].

By contrast, emerging evidence suggests that, in ageing instrumentalists, episodic memory effects occur without parallel enhancements in semantic verbal fluency [24, 25]. Instead, an association between instrumental activities and phonemic verbal fluency has been reported without parallel effects in episodic memory [24, 65]. Given the lexical-semantic component of singing and its absence in instrumental training, it seems possible that episodic memory benefits associated with singing [8, 16] versus instrumental activities are linked to partially different mechanisms.

As singing is currently underrepresented in neurocognitive research literature, however, with limited evidence on its effects on episodic memory [44, 46, 47], longitudinal evidence controlling for confounding factors including age, education, concurrent leisure activities, and cognitive status is needed to test this idea. Further, future studies should consider the choice of outcome measures since the underlying cognitive processes, also with respect to semantic versus episodic strategies, may differ between tests [70], potentially influencing the scores with respect to verbal fluency. Similarly, it is recommended to consider the use of retrieval strategies in verbal fluency tasks.

## Conclusion

The present study systematically mapped the associations of choir singing experience with well-being measures and cognitive performance with respect to the decades-long continuum of normal ageing. The study used a balanced sample (*N* = 95) of cognitively intact, healthy adults (aged 21–88 years) to map the co-occurrence and mediating mechanisms of these factors with respect to ageing, presenting choir singing experience by means of lifetime duration as well as highest (lifetime) and present frequency to address dose effects shown in previous works. Corroborating previous evidence, the results showed that present choir singing frequency was associated with reduced depressive symptoms, which also mediated trend-level associations with psychological and total QOL. Age-adjusted lifetime duration of choir singing was associated with enhanced performance in verbal fluency and episodic memory tasks (immediate, delayed) within the subgroup of choir singers and in the full sample, thus suggesting enhancements across the long age range. Here, semantic verbal fluency positively mediated the effects in episodic memory.

Despite the straightforward pattern of results, the present study is not without limitations. First, although measuring against the lifetime duration of choir singing can offer a sense of a longitudinal perspective, a cross-sectional design does not establish a causal relationship, nor does it provide ideal settings for testing mediation [36]. Conclusions drawn from a limited sample size (*N* = 95) also require caution, especially in the case of results with higher novelty value and hence less support from previous literature. Long-lasting RCTs, ideally involving multiple methods as well as recorded history of long-term engagement in leisure activities, are required to systematically test the effects of this cross-sectional exploratory study [71]. Second, self-selection of musical activities may lead to population bias through natural disposition for music. While lifetime-range correlational studies, such as the present one, can help map effects associated with various degrees of experience, controlling for musical aptitude as well as its biological underpinnings in a naturalistic sample is highly challenging. Higher reliability could be achieved through the standardisation of easily applicable control methods to test for known biological underpinnings of musical aptitude [72]. Finally, to our knowledge, the present study is the first of its kind in the context of singing and ageing, testing for multivariable effects across several different measures with a special interest in dose effects of singing. Lack of appropriate reference points on methodological choices, such as tests of episodic memory, hampers the generalisation of the observed effects. The present data does not rule out the possibility of contrasting results from other measures, measure combinations or a longitudinal design in future studies; ideally, future studies would indeed use partially different measures to extend the present findings.

These limitations considered, the present study corroborates previous works by showing, first, that choir singing is associated with reduced depressive symptoms, which also mediated trend-level enhancements of QOL. The pattern of results also suggested that the effects may be more prominent within choir singers, which would indicate group singing activities to promote well-being in an effective yet non-unique manner. Second, the present study extends previous findings by showing, for the first time, that singing-associated benefits on episodic memory are mediated by semantic verbal fluency. This contrasts previous evidence from instrumental activities, during which linguistic-semantic mapping does not automatically occur, and where, to our knowledge, benefits on semantic fluency and episodic memory have not been reported in parallel. Thus, the present study encourages not only the use of choir singing to promote a healthy ageing through enhanced well-being and memory performance, but also taking a closer look at the potentially multivariable mechanisms driving the positive impact of musical leisure activities in ageing, especially considering that ageing-related declines are seldom strictly separable from another.

## Data Availability

The data that support the findings of this study are available from the corresponding author upon reasonable request.
